# Material deprivation and rates of all-terrain vehicle- and snowmobile-related injuries in Ontario from 2003 to 2018: a population-based study

**DOI:** 10.17269/s41997-020-00416-0

**Published:** 2020-10-14

**Authors:** Alanna K. Chu, Trevor van Ingen, Brendan Smith, Sarah A. Richmond

**Affiliations:** 1 Division of Epidemiology, Dalla Lana School of Public Health, University of Toronto, 155 College St., Toronto, Canada; 2 Analytic Services, Public Health Ontario, 480 University Avenue, Toronto, Canada; 3 Health Promotion, Chronic Disease and Injury Prevention, Public Health Ontario, 480 University Avenue, Toronto, Canada; 4 Applied Public Health Science Unit, Health Promotion, Chronic Disease and Injury Prevention, Public Health Ontario, 480 University Avenue, Toronto, Canada

**Keywords:** Wounds and injuries, Socioeconomic factors, Off-road motor vehicles, Public health, Blessures, facteurs socioéconomiques, véhicules tout-terrain, santé publique

## Abstract

**Objectives:**

Socio-economic status (SES) is a well-established predictor of health outcomes; however, there is a dearth of evidence on the relationship between SES and off-road vehicle (ORV) injuries. In Ontario, all-terrain vehicles (ATVs) and snowmobiles present a serious risk for preventable injury. This study assessed the association between area-level material deprivation and the risk of ATV- and snowmobile-related injuries in Ontario, as well as the impact of sex and age.

**Methods:**

A population-based, repeat cross-sectional study was conducted using administrative data of ATV- and snowmobile-related emergency room visits from 2003 to 2018. Material deprivation was measured using the Ontario Marginalization Index, which assigned a score and quintile of deprivation to each dissemination area in Ontario. Age-standardized incidence rates and relative index of inequality values were calculated, stratified by quintile of deprivation, sex, age group, vehicle type, and health region.

**Results:**

We found a significant, positive relationship between ORV-related injuries and quintile of material deprivation (RII = 1.28, 95% CI: 1.01–1.63). Rates of ATV- and snowmobile-related injuries remained stable over time. Across all age groups, sex, and rural categories, we found an inverse u-shaped relationship between rates of injuries and quintile material deprivation. Males, individuals living in rural areas, and adolescents and young adults experienced the highest rates of injuries.

**Conclusion:**

Despite the positive relationship between ORV-related injuries and quintiles of deprivation, the inverse u-shaped relationship suggests that this increased risk of injury is likely related to exposure to ORVs. These results contribute to an understanding of the prevalence of the injury problem at a local level in Ontario. Stable rates of injury over time suggest that current public health programs are not sufficient in reducing these injuries, and further research should determine which factors amenable to intervention are contributing to increased risk of injury.

**Electronic supplementary material:**

The online version of this article (10.17269/s41997-020-00416-0) contains supplementary material, which is available to authorized users.

## Résumé

**Objectifs:**

Le statut socioéconomique (SSE) est un prédicteur bien établi des résultats pour la santé, mais il existe une pénurie de preuves sur la relation entre le SSE et les blessures des véhicules hors route. En Ontario, les véhicules tout-terrain (VTT) et les motoneiges présentent un risque sérieux de blessures évitables. Cette étude a évalué l’association entre la privation matérielle au niveau de la zone et le risque de blessures liées aux VTT et aux motoneiges en Ontario, ainsi que l’impact du sexe et de l’âge.

**Méthodes:**

Une étude transversale répétée basée sur la population a été menée à l’aide des données administratives des visites aux urgences liées aux VTT et aux motoneiges de 2003 à 2018. La privation matérielle a été mesurée à l’aide de l’Ontario Marginalization Index, qui a attribué un score et un quintile de privation à chaque aire de diffusion en Ontario. Les taux d’incidence normalisés selon l’âge et l’indice relatif des valeurs d’inégalité ont été calculés, stratifiés par quintile de privation, sexe, groupe d’âge, type de véhicule et région sanitaire.

**Résultats:**

Nous avons trouvé une relation positive significative entre les blessures liées aux VTT et le quintile de privation matérielle (RII = 1,28, IC à 95% : 1,01 – 1,63). Les taux de blessures liées aux VTT et aux motoneiges sont demeurés élevés au fil du temps. Dans tous les groupes d’âge, sexe et catégories rurales, nous avons trouvé une relation en forme de U inverse entre les taux de blessures et les quintiles de privation matérielle. Les hommes, les personnes vivant dans les zones rurales, les adolescents et les jeunes adultes ont connu les taux de blessures les plus élevés.

**Conclusion:**

Malgré la relation positive entre les blessures liées aux VTT et les quintiles de privation, la relation en forme de U suggère que ce risque accru de blessures est probablement lié à l’exposition aux VTT. Ces résultats contribuent à une compréhension de la prévalence du problème de blessures au niveau local en Ontario. Des taux stables de blessures au fil du temps suggèrent que les programmes de santé publique actuels ne sont pas suffisants pour réduire ces blessures, et des recherches plus poussées devraient déterminer quels facteurs susceptibles d’intervenir contribuent à augmenter le risque de blessures.

## Introduction

Socio-economic status (SES) has been recognized as an important predictor of both chronic and acute health outcomes (Lago et al. 2018; Stringhini et al. 2017). The relationship between SES and injury, however, is more complex and not well understood, particularly for non-fatal injuries (Cubbin & Smith 2002; Potter et al. 2005; Simpson 2005; Yuma-Guerrero et al. 2018). Previous research has found that the direction of the relationship between SES and injuries depends on numerous factors, including the population under study, cause of injury, type of injury, measure of SES, and the physical environment (Cubbin & Smith 2002; Potter et al. 2005; Simpson 2005). A recent systematic review examining measures of SES and injury outcomes in children and youth found that the majority of studies reported lower SES was associated with increased risk of unintentional injury, including road traffic injuries and burn and scald injuries, while other studies report higher SES associating with increased risk of injuries attributable to sport or recreation activities (Burrows et al. 2012; Simpson 2005; Zandy et al. 2019). There is insufficient literature in other injury topic areas, including few studies that examine fall- or drowning-related injuries or injuries due to the use of off-road vehicles.

In Ontario, off-road vehicles (ORVs) such as all-terrain vehicles (ATVs) and snowmobiles are popular recreational and transport vehicles; however, they present a risk of preventable injury with their use. According to the most recent Canadian Community Health Survey (CCHS) that collected information specific to ATV and snowmobile use in Ontario, over 20% of youth ages 12–15 years report using an ATV at least once in the previous 12-month period (Ontario Agency for Health Protection and Promotion (Public Health Ontario) et al. 2019). For snowmobile use, the number increases to over 28%. Given the size and weight of these vehicles, and the reported speed that they can travel (e.g., more than 90 km/h), there is a serious risk for injury, particularly in children and youth. In 2015 and 2016, there were 11,091 emergency department visits related to ATVs and 2944 related to snowmobiles, with the highest rates of injury reported among males, people 12 to 15 years of age, and those living in rural areas (Public Health Ontario, 2019). Several risk factors of ATV- and snowmobile-related injuries have been identified, such as driver and vehicle characteristics, use of protective gear, driving position, and driving patterns (Denning et al. 2014; Rodgers 2001); however, to date, no studies have investigated the relationship between SES and ORVs.

The purpose of this paper was to assess the association between area-level material deprivation and the risk of ATV- and snowmobile-related injuries in Ontario. Material deprivation is a measure of SES which is closely related to poverty and considers multiple domains such as housing, education, income, and employment. Area-level material deprivation is often used when individual-level measures of SES are unavailable. It has been shown to be strongly associated with health outcomes (Imlach Gunasekara et al. 2013; Pfoertner et al. 2011; Tøge & Bell 2016). The impact of sex and age on the risk of injuries related to ATV and snowmobile was also assessed. These results will lead to a better understanding of the nature of the relationship between SES and ORV-related injuries, and can be used to inform injury prevention priorities and interventions related to these vehicles.

## Methods

### Study population

We conducted a population-based, repeat cross-sectional study in Ontario, Canada, using administrative data collected from the provincial universal health care system. We used data from the National Ambulatory Care Reporting System (NACRS) database from the calendar year 2003–2018. NACRS contains visit records from 100% of emergency department visits in Ontario, Canada.

### Outcomes

ATV- and snowmobile-related injuries were defined as an emergency department visit related to an ATV or snowmobile identified in NACRS using the International Classification of Disease 10th Edition, Canada (ICD-10-CA) codes V86 and U99.032 codes (Supplementary Table 1). To assess the number of emergency department visits for ATV- and snowmobile-related injuries, only the emergency department visits associated with a unique NACRS visit key were included in the analysis, removing duplicate records from the same emergency department visit.

Material deprivation was measured using the Ontario Marginalization Index (ON-Marg). ON-Marg is an Ontario-specific version of the Canadian Marginalization Index, developed by Matheson et al. (2012). ON-Marg was created using principal component factor analysis at the dissemination area (DA) level on 18 Canadian census variables related to social and economic marginalization. Four factors were produced corresponding to four dimensions of marginalization: material deprivation, residential instability, ethnic concentration, and dependency. ON-Marg assigns each DA a marginalization score for each dimension. All Ontario DAs are further ranked and divided into quintiles each containing one fifth of the overall number of DAs, where quintile 1 contains the least marginalized DAs and quintile 5 contains the most marginalized DAs. It is derived based on indicators of income (proportion of adults unemployed, proportion of households considered low income, proportion of income from government transfer payments), quality of housing (i.e., proportion of households living in dwellings in need of major repair), low educational attainment (i.e., > high school diploma), and family structure characteristics (e.g., lone-parent families).

ON-Marg was used to assign each visit to an area-based measure of material deprivation at the DA level. The Statistics Canada Postal Code Conversion File Plus (PCCF+) version 7A was used to probabilistically geocode cases based on postal code of residence extracted from NACRS.

Postal code was also used to assign cases to public health units (PHUs). PHUs were further grouped into health unit regions: central east, central west, eastern, north east, north west, south west, and Toronto (Supplementary Table 2). For each ATV- and snowmobile-related injury, the patient’s age and sex were extracted from the NACRS database. Age was grouped into three categories (0–14, 15–29, 30+ years), and sex was categorized into female and male.

### Statistical analyses

Rates of all ORV injuries occurring between 2003 and 2018 were age-standardized to the 2011 Canadian population and stratified by quintile of material deprivation. The 2006 version of ON-Marg was used to assign quintiles of material deprivation for injuries occurring in 2003 to 2010, while the 2016 version of ON-Marg was used for injuries occurring in 2011 to 2018. Census DA profiles were used to calculate denominators for each quintile for the 2006 and 2016 populations, and linear extrapolation was used to estimate denominators for the remaining calendar years. Additional subgroup analyses were conducted stratifying injuries across quintiles and time, sex (female, male), age group (0–14, 15–29, 30+ years), injury type (ATV, snowmobile), and health region (central east, central west, eastern, north east, north west, south west, Toronto).

To quantify differences in rates of injuries across quintiles of marginalization, the relative index of inequality (RII) values were calculated for all analyses. The RII is a regression-based measure that takes into account differences in rates across all five quintiles of marginalization. The RII was calculated by fitting a multiplicative Poisson model based on the work of Moreno-Betancur et al. (2015). Relative index of inequality values of greater than one indicate that risk of injury is higher in the most marginalized areas compared with the least. Statistical significance occurs where 95% confidence intervals (95% CIs) do not include the null value of 1. Only those cases that could be assigned to a level of marginalization were included in these results. A total of 115,628 cases were successfully extracted and 4200 were excluded (648 missing postal code, 409 PCCF+ unable to geocode legitimate Ontario DAs, and 3143 assigned to DAs with no ON-Marg).

Using methodology developed by Health Quality Ontario (2019), data from 2014 to 2018 were stratified by urban-rural categories, using 2016 census population data multiplied by 5 for the denominator, to produce age-standardized incidence rates and RII values. Only data from 2014 to 2018 were used, as rural-urban category data were only available for 2016 census geographies. Rates were age-standardized to the 2011 census.

This study received research ethics approval from the Public Health Ontario Research Ethics Board.

## Results

### Off-road vehicle injuries and material deprivation in Ontario

Of the 111,482 off-road vehicle injuries included in this study, 84,971 were ATV-related and 26,511 were snowmobile-related. The age-standardized incidence rate (ASIR) of ORV-related injuries in Ontario for the years 2003 to 2018 was 55.4 per 100,000 population, the ASIR of ATV-related injuries was 42.2 per 100,000, and the ASIR of snowmobile injuries in Ontario was 13.2 per 100,000 population.

**Fig. 1:**
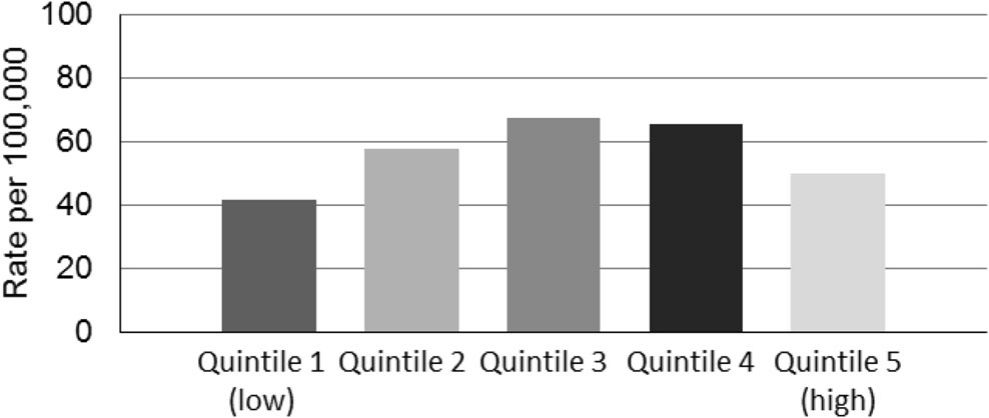
Off-road vehicle-related age-standardized emergency department visit rate per 100,000 population, by quintile of material deprivation in Ontario for 2003 to 2018

Figure 1 shows the ASIR of ORV-related injuries per 100,000 population. Across the entire study period, the rate of ORV-related injuries was the highest in the third quintile of material deprivation (67.5 per 100,000), followed by the fourth quintile (65.6 per 100,000), second quintile (57.8 per 100,000), fifth quintile (49.7 per 100,000), and finally first quintile (41.6 per 100,000). Despite the relatively low rates found in highest quintile of material deprivation, the RII of 1.28 (95% CI: 1.01–1.63) indicates a significant association between material deprivation and ORV-related injuries in Ontario (Table 1).

**Table 1:** Off-road vehicle-related age-standardized emergency department visit rate per 100,000 population, and relative index of inequality (RII) measuring the association with quintile of material deprivation for analyses of all of Ontario, by sex, age group, injury type, region of Ontario, and urban-rural category

Analysis		ASIR per 100,000	RII	95% CI of the RII
Ontario		55.38	1.28	(1.01–1.63)
Sex^1^	Female	24.22	1.50	(1.21–1.87)
	Male	87.14	1.23	(0.96–1.57)
Age group^1^	0 to 14	49.67	1.15	(0.5–2.63)
	15 to 29	122.30	1.21	(0.61–2.37)
	30 +	35.53	1.35	(0.8–2.26)
Injury type^1^	ATV	42.17	1.26	(0.99–1.59)
	Snowmobile	13.20	1.35	(1.04–1.76)
Health region^1^	Central east	46.70	1.41	(1.29–1.54)
	Central west	51.34	1.10	(0.84–1.44)
	Eastern	76.59	2.17	(1.57–2.99)
	North east	192.02	1.15	(1.02–1.31)
	North west	140.96	0.82	(0.68–0.99)
	South west	88.90	0.83	(0.61–1.13)
	Toronto	6.13	0.42	(0.35–0.49)
Urban-rural category^2^	Large urban community	15.42	0.71	0.64–0.79
	Med/small in CMA/CA*	88.63	1.43	1.22–1.67
	Rural in CMA/CA	159.47	1.49	1.37–1.63
	Rural/remote	224.45	1.22	1.07–1.39

*Census Metropolitan Area/Census Agglomeration

^1^Results reflect data collected between 2003 and 2018, age-standardized to the 2011 Canadian census; data from 2003 to 2010 were assigned to quintiles of material deprivation from the 2006 ON-Marg; and data from 2011 to 2018 were assigned using the 2016 ON-Marg

^2^Results reflect average rates between 2014 and 2018, age-standardized to the 2011 Canadian census; data were assigned using the 2016 ON-Marg

### Inequities in off-road vehicle injury over time

**Fig. 2:**
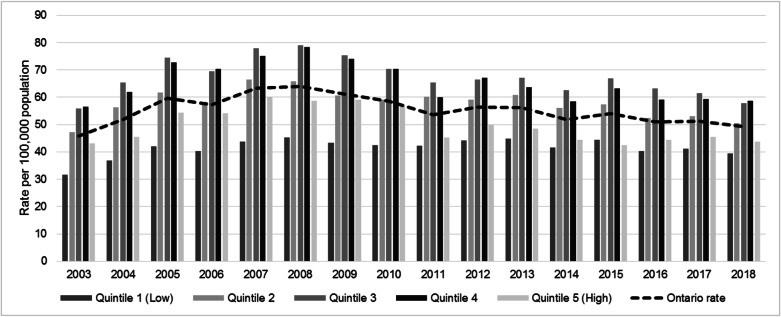
Off-road vehicle-related age-standardized emergency department visit rate per 100,000 population, by quintile of material deprivation in Ontario and by calendar year for 2003 to 2018

**Fig. 3:**
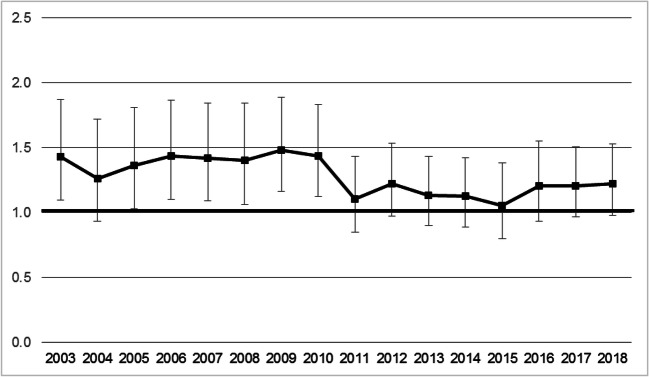
Relative index of inequality (RII) for material deprivation on off-road vehicle injuries in Ontario from 2003 to 2008, 2014–2017

Figure 2 illustrates the distribution of rates of ORV-related injuries by quintile of material deprivation for the years 2003 to 2018. There is an increase in the rate of injuries from 45.8 per 100,000 in 2003 to 64.0 per 100,000 in 2008, followed by a steady decline to 49.3 per 100,000 in 2018. Across all years, the first and fifth quintiles have the lowest rates of ORV-related injuries, and the second, third, and fourth quintiles have the highest rates of ORV. As shown in Fig. 3, statistically significant RIIs of greater than one were observed for the years 2003 and 2005–2010.

### Inequities in off-road vehicle injury by age group

**Fig. 4:**
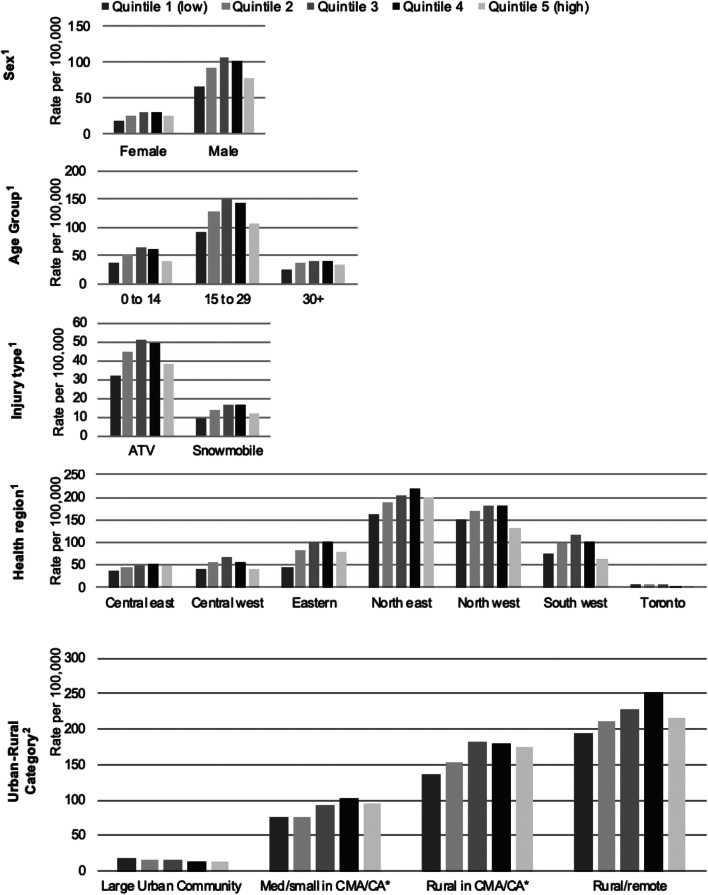
Off-road vehicle-related age-standardized emergency department visit rate per 100,000 population, by quintile of material deprivation in Ontario for 2003 to 2018 by sex, age group, injury type, and region of Ontario. *Census Metropolitan Area/Census Agglomeration. ^1^Results reflect data collected between 2003 and 2018, age-standardized to the 2011 Canadian census; data from 2003 to 2010 were assigned to quintiles of material deprivation from the 2006 ON-Marg; and data from 2011 to 2018 were assigned using the 2016 ON-Marg. ^2^Results reflect average rates between 2014 and 2018, age-standardized to the 2011 Canadian census; data were assigned using the 2016 ON-Marg

The ASIR of ORV-related injuries per 100,000 population in 2003–2018 was 49.7 among 0 to 14 year olds, 122.3 among 15 to 29 year olds, and 35.5 among those aged 30 and older (Table 1). Similar trends by quintile of material deprivation exist across all age groups, where the ORV injury rate was the highest rate for the third quintile, while the lowest rates were found in the first quintile, followed by the fifth quintiles of material deprivation (Fig. 4). Non-significant RII results were found for all age groups (Table 1).

### Inequities in off-road vehicle injury by sex

The ASIR of ORV-related injuries per 100,000 population in 2003–2018 was 87.1 for males and 24.1 for females (Table 1). Males in the third quintile had the highest rate of ORV-related injuries, while the lowest rates were found in the first quintile, followed by the fifth quintile of material deprivation. In females, the highest rates were found in the fourth quintile, and the lowest rates were found in the first quintile, followed by the fifth quintile of material deprivation (Fig. 4). The RII indicates statistically significant inequities where material deprivation is positively associated with ORV injury for females (RII: 1.50, 95% CI: 1.21–1.87) but not for males (RII: 1.23, 95% CI: 0.96–1.57) (Table 1).

### Inequities in off-road vehicle injury by injury type

The ASIR per 100,000 population was higher for ATV-related injuries (42.2) than for snowmobile-related injuries (13.2) in 2003–2018 (Table 1). ATV-related injury rates were highest in the third quintile, while the lowest rates were found in the first quintile, followed by the fifth quintile of material deprivation (Fig. 4). Snowmobile-related injury rates were highest in the third and fourth quintiles, while the lowest rates were found in the first quintile, followed by the fifth quintile of material deprivation (Fig. 4). The RII indicates statistically significant inequities where material deprivation is positively associated with snowmobile-related injury (RII: 1.35, 95% CI: 1.04–1.76) but not with ATV-related injury (RII: 1.26, 95% CI: 0.99–1.59) (Table 1).

### Inequities in off-road vehicle injury by region of Ontario

The ASIR for ATV- and snowmobile-related injuries varied across regions in Ontario. They were highest among the northern regions of Ontario (north east and north west) and lowest among individuals living in Toronto (Fig. 4). Material deprivation was positively associated with ORV-related injuries for the central east, eastern, and north east regions, null relationships in the central west and south west regions, and inverse relationships for the north west and Toronto regions (Table 1). It is important to note that ON-Marg scores are not available for DAs where complete census data are not available; this happens most often in rural areas and reserves. While in most regions less than 2% of cases cannot be assigned to an ON-Marg quintile, this increases to 6.2% of cases residing in the north east region, and 13.2% of cases residing in the north west region.

### Inequities in off-road vehicle injury by rural-urban category in Ontario

The ASIR for ATV- and snowmobile-related injuries varied across rural-urban categories in Ontario (Fig. 4). The highest rates were in rural/remote areas, followed by rural areas in Census Metropolitan Area (CMA)/Census Agglomeration (CA), then medium/small areas in CMA/CA, and finally large urban communities. Across quintiles of deprivation, the third and fourth quintiles represented the highest rates, followed by the fifth quintile, second quintile, and first quintile, respectively, for all the rural-urban categories except large urban communities. The RII indicates significant inequities where material deprivation is positively associated with off-road vehicle injuries in all rural categories (Table 1).

## Discussion

This study is the first to examine the relationship between material deprivation and ORV-related injuries in Ontario, Canada. Our results show an inverse U relationship across quintiles of material deprivation with relatively low rates of ORV injury in the highest quintile of deprivation. Our results show that the rates of ORV-related injuries remain stable over time and that there is a pronounced sex difference, where males experience a higher rate of ORV-related injuries compared with females. Rates of ORV-related injury were notably higher in adolescent and young adults, in the north east and north west public health regions, and in rural/remote areas, and the overall rate was similar to rates of pedestrian injuries in Ontario (Parachute 2018). We found higher rates in increasingly rural areas and a positive and significant relationship between ORV-related injuries and quintile of material deprivation within all rural categories. The higher rates in rural areas and a lack of association within the large urban community category suggest that the major risk factor for ATV- and snowmobile-related injuries may simply be exposure to these vehicles. Across all age groups, sex, and regions, rates of injury were highest among the middle quintile of material deprivation and lower among the highest and lowest quintiles, indicating that the middle-income group is at the highest risk for ORV-related injuries. It is possible that those in the highest quintile of material deprivation are not exposed to ORVs due to lack of access to these vehicles.

These results are important to inform a program of public health that includes understanding the prevalence of the injury problem at a local level, as well as the contributing factors that may play a role in the risk of sustaining an injury. These data demonstrate that the rate of injury may not be associated with material deprivation but may be related to ORV use which is relatively stable over time. These data imply that, while the rate of injury is not increasing, our current efforts to reduce injuries related to ORV use are insufficient. Our data also demonstrate the highest rate of injury was found in adolescents and young adults, and in rural areas over urban areas, as well as in the Q3 and Q4 SES quintiles—or those that are associated with the middle-income groups.

Given the consistent rate of injury to adolescents and young adults from ORV use in Ontario, implementation of policy-level interventions that aim to reduce exposure to ATVs and snowmobiles should be considered in a comprehensive program of public health. Prior research has shown that children do not have the physical or cognitive capacity to safely operate these motor vehicles, particularly in unpredictable environments (Anson et al. 2009; Denning et al. 2014). ATVs have a high centre of gravity, and children and youth do not have the weight or strength to control the vehicle, leading to a high risk of rolling the vehicle (Mattei et al. 2011). Rolling of the vehicle can cause significant injury, particularly to children and youth, given the weight and size of ATVs. Legislation mandating age restrictions and graduated driver licensing, similar to licensing of other motor vehicles, should be considered to prevent exposure to these vehicles among individuals who are too young to operate them safely. These strategies have been shown to be effective in reducing on-road motor vehicle injuries in young people (McKnight & Peck 2002).

### Strengths and limitations

This is the first study to examine measures of SES with ORV-related injuries in Canada. This study used population-level data sources to report the rate of emergency department visits related to ORV use, by age and sex—an update to the literature in this area. In addition, we report on summary measures of SES, including the report of RII, which describes differences between material deprivation quintile in a population relative to the population mean. This measure is most useful to study changes over time.

This study is subject to a number of limitations. First, the data are limited to emergency department visits related to ORVs and thus are likely an under-representation of the total burden of ATV- and snowmobile-related injuries. These data do not include injuries not treated in the emergency department. We also identified ATV- and snowmobile-related injuries using the ICD-10 code V86; this code may include injuries related to other off-road vehicles (e.g., golf carts, dirt bikes); therefore, these results may include emergency department visits related to other off-road vehicles. Further, ICD-10 coding includes report of injuries associated with the home address of the individual presenting to hospital, as opposed to where the injury actually occurred; therefore, it is possible that injuries reported within a region in Ontario may have actually occurred in another area. It is estimated, however, that the majority of ORV use corresponds with the location of the reported injury.

Material deprivation as the measure of SES used in this study may not consider other aspects of socio-economic status which may be important predictors of risk of injury. We also used area-level measures of material deprivation and urban/rural categories, not individual-level data, which can be subject to ecological fallacy. Additionally, urban-rural categories may not account for differences that may affect ORV engagement within categories. In this case, the associations observed across groups of individuals may not truly represent the association observed at an individual level. In addition, the relationship between SES and injury was assessed using a cross-sectional analysis. This analysis does not account for individual changes in SES over time.

Finally, the measure of RII was used to discuss the changes in SES over time. These results are derived from log-linear Poisson regression models that are conditional on meeting the assumptions of linear regression analyses. Specifically, there is an assumption that the change in injury risk across all quintiles of SES is linear on a log scale. Where these assumptions are false, the use of RII may not be appropriate. Future studies should use a multifactorial and multilevel approach to analyze the effect of SES on the risk of injury.

Another limitation of the study is that not all regions have equal representation on the ON-Marg, as ON-Marg values cannot be produced where census data are not available for all indicators. This happens most often in reserves and rural areas, and the north west region has a particularly high proportion of the population that cannot be assigned ON-Marg scores. This could possibly bias the regional results.

Last, future studies should examine access to ORVs compared with rates of injury as well as purpose of use (i.e., recreational, transportation, and/or vocational), in order to determine whether or not risk of injury is related to exposure to vehicles and in what context. Furthermore, a greater understanding of the attitudes and motivation towards engaging with ORVs would inform directionality of public health initiatives.

## Conclusion

Our results found evidence to suggest that material deprivation was associated with ORV-related injuries. However, stratification of the data demonstrates that rates of ORV-related injuries are highest among males as compared with females, in adolescents and young adults, and in rural/remote areas of Ontario as compared with urban areas. Public health policy interventions should aim to reduce exposure to ATVs and snowmobiles among younger drivers.

## Electronic supplementary material

ESM 1 (DOCX 19 kb)
