# Supplementary material for: Material deprivation and rates of all-terrain vehicle- and snowmobile-related injuries in Ontario from 2003 to 2018: a population-based study
Source: Can J Public Health. 2020 Oct 14;112(2):304–12. doi: 10.17269/s41997-020-00416-0 (PMC7910353; doi:10.17269/s41997-020-00416-0)
Supplement: Supplementary file 1 — (DOCX 19 kb) [file 41997_2020_416_MOESM1_ESM.docx]

**Supplementary Table 1.** International Classification of Disease (ICD-10 Canadian Version) codes for cause of injury from off-road vehicles

| Off Road Vehicle | ICD-10 Codes |
| --- | --- |
| Snowmobile | Highway: V8600, V8610, V8630  Non-Highway: V8650, V8651, V8660, V8661, V8690, V8691 |
| All-Terrain Vehicle | Highway: V8608, V8618, V8638, V862  Non-Highway: V8658, V8668, V8698, V867  Other: V864, U99 |

**Supplementary Table 2.** Ontario Health Unit Regions

| **Health unit region** | **Public Health Units** |
| --- | --- |
| Central East | Durham Region Health Department,  Haliburton, Kawartha, Pine Ridge District Health Unit,  Peel Public Health,  Peterborough Public Health,  Simcoe Muskoka District Health Unit,  York Region Public Health |
| Central West | Brant County Health Unit,  City of Hamilton Public Health Services  Haldimand-Norfolk Health Unit  Halton Region Public Health  Niagara Region Public Health  Region of Waterloo Public Health and Emergency Services  Wellington-Dufferin-Guelph Public Health |
| Eastern | Hastings Prince Edward Public Health  Kingston, Frontenac and Lennox & Addington Public Health  Leeds, Grenville and Lanark District Health Unit  Ottawa Public Health  Renfrew County and District Health Unit  Eastern Ontario Health Unit |
| North East | Algoma Public Health  North Bay Parry Sound District Health Unit  Porcupine Health Unit  Public Health Sudbury & Districts  Timiskaming Health Unit |
| North West | Northwestern Health Unit  Thunder Bay District Health Unit |
| South West | Chatham-Kent Public Health  Grey Bruce Health Unit  Huron County Health Unit  Lambton Public Health  Middlesex-London Health Unit  Perth District Health Unit  Southwestern Public Health  Windsor-Essex County Health Unit |
| Toronto | Toronto Public Health |
